# Knocking on the Doors of Perception: the role of psilocybin in substance use disorder treatment

**DOI:** 10.1192/j.eurpsy.2023.613

**Published:** 2023-07-19

**Authors:** R. Sousa, L. Costa, J. Brás, R. Vaz, J. Martins, J. Abreu, E. Almeida, N. Castro, R. Andrade, N. Cunha

**Affiliations:** ^1^Departamento de Psiquiatria e Saúde Mental, Centro Hospitalar Tondela-Viseu; ^2^Centro de Respostas Integradas Viseu, Divisão de Intervenção em Comportamentos Aditivos e Dependências, ARS Centro, Viseu, Portugal

## Abstract

**Introduction:**

Substance use disorders(SUDs) are a major health concern and current treatment interventions have proven only limited success. Despite increasing effectiveness, still about 50–60% relapse within 6–12 months after treatment [Cornelius *et al*., Addict Behav. 2003;28 381-386]. SUDs are defined as chronic disorders of brain reward system, motivation, and memory processes that have gone awry. Medication reducing craving and substance use is mainly available for alcohol dependence and to a lesser extent for other substances.

Hallucinogens may represent a group of agents with potential anti-craving properties subsequently reducing substance use in SUD patients. For instance, lysergic acid diethylamide(LSD) and psilocybin have previously been shown to effectively alleviate symptoms of alcohol and nicotine dependence.

**Objectives:**

New treatments preferably focusing on reducing craving and subsequent substance use are therefore urgently needed. The hallucinogen psilocybin may provide a new treatment option for SUD patients, given the beneficial results observed in recent studies

**Methods:**

Systematic revision of literature.

**Results:**

In the 1950s, a group of drugs with potential to alter consciousness were discovered (hallucinogens). Several studies suggested their anti-SUD potential, improving self-acceptance and interpersonal relationships, reducing craving and alcohol use. As a result of its recreational popularity during the 1960s, they were banned in 1967, greatly hampering scientific research in this field. Recently, psilocybin, an hallucinogenic substance in psilocybin-containing mushrooms has gained popularity in neuropsychological research, showing to increase trait openness, cognitive and behavioral flexibility, and ratings of positive attitude, mood, social effects, and behavior and even reported persistent positive changes in attitude and behavior. These findings might suggest a valuable compound for the treatment of psychiatric conditions with several additional studies providing supportive evidence for the therapeutic potential of psilocybin for SUD treatment and relapse prevention.

**Conclusions:**

With the reported limited amount of side effects and potential beneficial effects of psilocybin in SUD, there are valid reasons to further investigate the therapeutic efficacy and safety of psilocybin as a potential SUD treatment. On the one hand, psilocybin may exert its anti-addictive properties by beneficial effects on negative emotional states and stress. On the other hand, psilocybin may improve cognitive inflexibility and compulsivity. Research on the efficacy of psilocybin on SUD is still limited to a handful of published studies to date. As a result, many important questions related to the use of psilocybin as a complement to current treatment of SUD and its working mechanisms remain unanswered. Before psilocybin can be implemented as a treatment option for SUD, more extensive research is needed.

**Disclosure of Interest:**

None Declared

